# Laboratory prognostic factors for the long-term survival of multiple system atrophy

**DOI:** 10.1038/s41531-022-00413-9

**Published:** 2022-10-27

**Authors:** Jung Hwan Shin, Han-Joon Kim, Chan Young Lee, Hee Jin Chang, Kyung Ah Woo, Beomseok Jeon

**Affiliations:** 1 Department of Neurology, Seoul National University Hospital & Seoul National University College of Medicine, Seoul, South Korea; 2 Department of Neurology, School of Medicine, Ewha Womans University Mokdong Hospital, Seoul, South Korea

**Keywords:** Movement disorders, Predictive markers

## Abstract

To elucidate the biomarkers related to survival in multiple system atrophy(MSA), we analyzed the predictability of retrospectively collected blood markers for survival in 650 probable MSA. High absolute neutrophil count, red-cell distribution width, C-reactive protein, erythrocyte sedimentation rate, and low hemoglobin, protein, albumin, and creatinine were correlated with higher mortality in MSA. Systemic alteration in inflammation and nutritional status in the early stage are associated with higher mortality in MSA.

Multiple system atrophy (MSA) is a rapidly progressive neurodegenerative disease with a mean survival of 6–10 years from disease onset^1^. Thus, it is important to establish a biomarker that predicts the prognosis in the early phase of the disease to optimize disease monitoring and to develop novel neuroprotective strategies.

Among clinical biomarkers, early autonomic failure, older age of onset, and absence of response to levodopa have been associated with shorter survival^2–4^. In laboratory biomarkers, there is growing evidence that biomarkers reflecting high systemic inflammation are associated with disease severity and progression of MSA^5,6^. Furthermore, recent studies revealed that biomarkers associated with neuronal damage^7^ and malnutrition^8^ are associated with disease severity, low quality of life, and high mortality in MSA. However, the role of multiple peripheral blood markers reflecting systemic inflammation, and metabolic and nutritional state in the prediction of mortality has not been validated in a large number of MSA populations. Thus, we aimed to analyze the laboratory markers from the early stage of MSA as prognostic biomarkers for all-cause mortality.

A total of 650 probable MSA patients were enrolled (371 male and 279 female, baseline age 64.0 ± 8.7 years, disease duration = 2.7 ± 3.7 years, 366 MSA-, and 284 MSA-C). About 372 patients were deceased at the time of data collection and the median survival duration [95% confidence interval] from the onset was 8.0[7.5–8.5] years (Fig. 1A). Among 650 participants, 53 patients had no laboratory results and therefore, were excluded from further analysis. The median survival duration [95% confidence interval] of these 53 patients was 8.0[7.4–8.6] years (Fig. 1B) without statistical significance between MSA with laboratory results (log-rank, *p* = 0.27). The median survival of MSA-P patients and MSA-C patients were 9.0[8.3–9.7] and 8.0[7.4–8.6] years, respectively, with no statistical difference between the two subtypes.

**Fig. 1:**
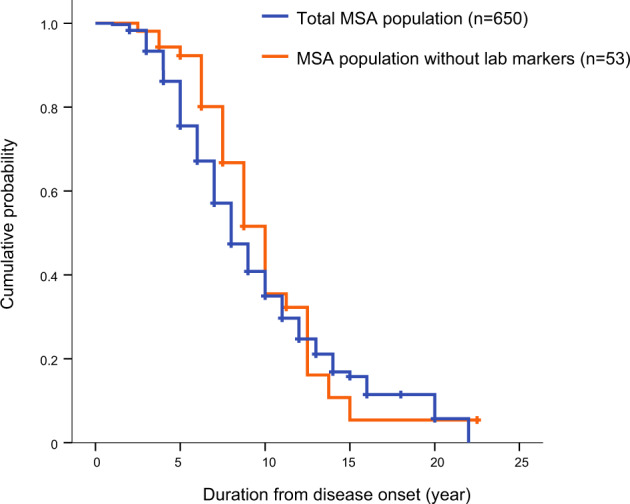
Disease-free survival in MSA patients with Kaplan–Meier analysis. Kaplan–Meier survival curve for total probable MSA (blue, *n* = 650) and MSA patients without any blood laboratory markers during follow-up (orange, *n* = 53).

The results of collected laboratory markers are summarized in Supplementary Tables 1, 2. All markers were normally distributed. The percentage (number) of MSA participants with available laboratory markers included in this study ranged from 36% (232) to 72% (465). The mean duration between motor symptom onset to the laboratory tests ranged from 3.42–4.29 years. The mean duration from the initial visit to the neurology clinic to the laboratory results ranged from 1.30–1.84 years.

With a cut-off of >mean value of laboratory markers within MSA patients, high WBC, ANC, RDW, CRP, and ESR values were significantly related to higher mortality. Low (<mean) lymphocyte, Hb, total protein, albumin, creatinine, calcium, and GPT were related to higher mortality (Fig. 2A). With a cut-off of > mean + 1 SD, high ANC, RDW, CRP, ESR, BUN, GFR values, and low albumin predicted higher mortality (Fig. 2B). With cut-off of > mean – 1 SD, high ESR and low lymphocyte, Hb, protein, albumin, creatine, and calcium predicted a worse outcome (Fig. 2C).

**Fig. 2:**
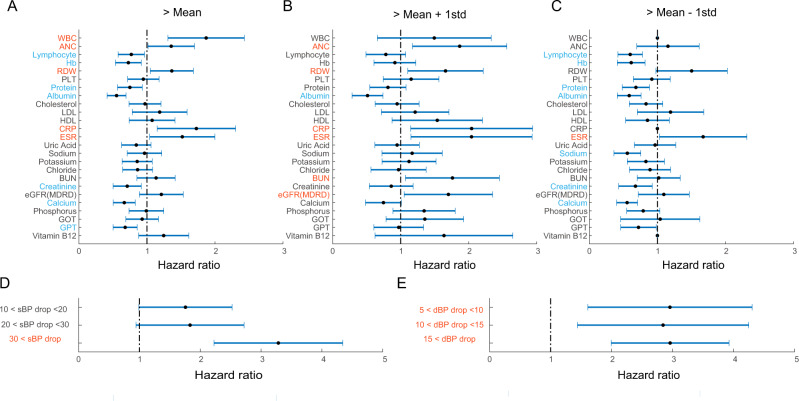
The hazard ratio of blood biomarkers in the prediction of survival in multiple system atrophy. **A** Bar graph of hazard ratio ± 95% confidence interval from each blood laboratory marker with a cut-off of >mean predicting mortality from longitudinal follow-up of MSA patients. The hazard ratios with statistical significance (*p* < 0.05) were marked as red (positive) or light blue (negative). A vertical dotted line denotes a hazard ratio of 1. **B** The hazard ratio for blood laboratory markers with a cut-off of >mean + 1 standard deviation. **C** The hazard ratio for blood laboratory markers with a cut-off of >mean − 1 standard deviation. There was no event (death) in MSA patients with less than the mean – 1 standard deviation of WBC, CRP, and Vitamin B12. **D**, **E** Bar graph of hazard ratio ±95% confidence interval of systolic blood pressure (sBP) drop (D) and diastolic blood pressure (dBP) drop in the orthostatic blood pressure test. Hazard ratio for sBP was calculated comparing MSA groups of [10 < sBP drop < 20 versus sBP < 10] and [20 < sBP drop < 30 versus sBP < 20], [30 < sBP versus sBP < 30]. Hazard ratio for dBP was calculated comparing MSA groups of [5 < dBP drop < 10 versus dBP < 5] and [10 < dBP drop < 15 versus dBP < 10], [15 < dBP versus dBP < 15]. std standard deviation, WBC white blood cell count, ANC absolute neutrophil count, Hb hemoglobin, RDW red-cell distribution width, PLT platelet count, LDL low-density lipoprotein, HDL high-density lipoprotein, CRP C-reactive protein, ESR erythrocyte sedimentation rate, BUN blood urea nitrogen, eGFR estimated glomerular filtration rate, GOT glutamic oxaloacetic transaminase, GPT glutamic pyruvic transaminase.

In orthostatic hypotension, sBP drop >30 significantly predicted higher mortality in MSA patients. MSA groups showing dBP drop of more than 5 mmHg significantly predicted higher mortality compared to a lesser dBP drop group (Fig. 2D, E).

In the MSA subtype, high (>mean) RDW and low (<mean) Hb, creatinine, and GPT significantly predicted higher mortality in MSA-P, not in MSA-C. High (mean) CRP and low (<mean) lymphocytes significantly predicted higher mortality in MSA-C, not in MSA-P. (Supplementary Fig. 1).

The current study presents novel findings regarding high inflammation and poor nutritional status associated with higher mortality in MSA.

Neuroinflammation is suggested as one of the pathomechanisms in MSA that affects core pathological processes, including propagation and aggregation of misfolded a-synuclein, oxidative stress, and mitochondrial dysfunction^9^. Previous studies have reported high inflammatory cytokines and markers in peripheral blood or CSF that correlates with disease severity^5,6,10^. In line with these results, we found high laboratory markers of WBC, ANC, ESR, and CRP, which reflect inflammation predicted higher mortality in the MSA population. A recent report showed that the neutrophil to lymphocyte (NRL) ratio significantly predicted higher mortality in MSA^11^, which corresponds with our data showing high (>mean) NRL predicted higher mortality (HR = 1.80[1.34, 2.42]).

Interestingly, we found that the higher RDW predicted higher mortality in MSA. Previous studies reported the association of high RDW value with high mortality in the general population^12,13^, heart failure^14^, cerebral infarction^15^, and cancer. Cross-sectional studies showed that RDW is higher in Parkinson’s disease (PD) when compared to healthy controls and correlates with disease severity^16,17^. The exact mechanism of how elevated RDW is related to higher mortality, remains unclear. However, previous studies showed that inflammation or oxidative stress may inhibit the maturation of erythrocytes by suppressing the bone marrow, which results in increased RDW^18^.

Malnutrition is associated with higher mortality in the elderly population^19^ and MSA patients are frequently associated with weight loss^20^ and a malnutritional state^8^. In a previous cross-sectional study, daily calorie intake was decreased in MSA and was correlated with impaired daily activity^8^. Also, serum albumin showed a negative association with functional status in MSA^8^. In this study, we showed that low albumin, protein, and hemoglobin, which reflect poor nutritional status^21^, were associated with higher mortality in MSA. Interestingly, low creatinine level was associated with higher mortality in MSA in our data. Given that serum creatinine levels reflect muscle mass and renal function^22^, low creatinine levels in MSA may reflect muscle wasting in MSA and thus may contribute to impaired activities of daily living (ADL) and higher mortality. These observations suggest that close observation of the nutritional state from an early stage of the disease would be important in the prognosis of MSA^23^.

Of note is that the high RDW and low creatinine level significantly predicted higher mortality in MSA-P and not in MSA-C, which may suggest that subtype-specific (MSA-P vs MSA-C) contribution to inflammation and nutritional status in MSA.

In orthostatic blood pressure drop, sBP drop >30 mmHg from the second consensus criteria^24^ for MSA significantly predicted higher mortality, whereas sBP drop range from 20 to 30 mmHg, which can be defined as significant by the newly proposed MSA diagnostic criteria^25^ did not predict higher mortality. Moreover, a decrease in diastolic blood pressure, even in a lower range compared to the MSA diagnostic criteria, significantly predicted higher mortality compared to groups with lesser dBP drop group which corresponds with the previous studies showing the significantly higher impact of diastolic BP drop compared to systolic BP drop in the all-cause mortality in the MSA patients^26^. These patterns were found in both groups of MSA-P and MSA-C (Supplementary Fig. 1).

There are several limitations to this study. First, MSA patients in this study were not pathologically confirmed. Second, comorbidities of MSA patients, which may affect overall survival (e.g., hypertension, diabetes), were not included in this study. Also, disease severity, body mass index, or medications were not included as cofactors. Thus, there is a possibility that the hazard ratio of each marker in our study may have been biased. Third, from a retrospective nature, the reason for the peripheral blood test is variable among participants. There is a possibility that those who have undergone blood tests might be in poor medical condition, including infection. To avoid this, we excluded laboratory results from the emergency room and we chose the first laboratory test from the initial visit to the neurology clinic. We further analyzed the hazard ratios with the laboratory data that were collected within a year from the first visit, which resulted in overall similar results (Supplementary Fig. 2 and Supplementary Table 3). The mean CRP value in our MSA group was 1.75 mg/L, which was comparable with the previously reported CRP values from PD patients without any infection (1.53 mg/L)^27^. Also, the fact that the mean survival in patients without any laboratory tests (*n* = 53) was comparable with MSA patients with laboratory tests argues against the possibility that blood tests were more likely to be performed in patients with a poor medical condition. Nevertheless, our data requires cautious interpretation and future prospective design studies are required. In conclusion, a systemic alteration in inflammation and nutritional status was associated with a poor prognosis in MSA. Thus, close observation of inflammation and nutritional state from an early stage of the disease would be important in the prognosis of MSA.

## Methods

We retrospectively reviewed the medical records of patients who were diagnosed with probable MSA according to the second consensus criteria and were followed at the Movement Disorders Clinic in Seoul National University Hospital from 2011 to 2020. The diagnosis was based on the last evaluation. For clinical information, we collected age, sex, disease duration, and the onset of motor symptoms at the initial visit. The type of MSA was determined according to the predominant motor symptoms at the last visit: parkinsonism dominant (MSA-P) or cerebellar dominant (MSA-C).

We retrospectively collected the first laboratory data after the initial visit to the neurology clinic at Seoul National University Hospital. The laboratory tests that were obtained from the emergency room or with an emergency order, were excluded. The laboratory markers included white blood cell count (WBC), absolute neutrophil count (ANC), absolute lymphocyte count, hemoglobin (Hb), red-cell distribution width (RDW), platelet count (PLT), serum total protein, albumin, total cholesterol, low-density lipoprotein (LDL), high-density lipoprotein (HDL), serum uric acid, serum electrolyte, blood urea nitrogen (BUN), creatinine, glomerular filtration rate (GFR), calcium, phosphorus, vitamin B12, glutamic oxaloacetic transaminase (GOT), glutamic pyruvic transaminase (GPT), C-reactive protein (CRP), and erythrocyte sedimentation rate (ESR). We also retrospectively collected the orthostatic blood pressure tests. Maximal systolic and diastolic blood pressure drop within 3 min after standing was defined as sBP and dBP drop, respectively. The MSA patients were enrolled regardless of medications used for orthostatic hypotension. We collected the survival information as of August 2020 from the National Health Information Database in South Korea. The institutional review board of Seoul National University Hospital approved this study (2005-165-1125) and informed consent was waived due to the retrospective nature of the study.

Cox proportional hazards analysis adjusting for age, sex, and disease duration was used to calculate the hazard ratio for each laboratory marker. Kaplan–Meier survival analysis was used to draw a survival curve. The age and disease duration in the survival analysis was calculated from the time of the laboratory test. The cut-off for each variable was selected as >mean, >mean + 1 SD, and >mean – 1 SD within the MSA group. Kolmogorov–Smirnov test was used to verify the normal distribution of the laboratory parameters. Data were presented as mean ± standard deviation if normally distributed. All statistical analysis was performed using custom-written code in Matlab 2020a (MathWorks).

### Reporting summary

Further information on research design is available in the Nature Research Reporting Summary linked to this article.

## Supplementary information

Supplementary materials

Reporting Summary

## Data Availability

The datasets and code generated during the current study are available from the corresponding author on reasonable request, including reproducibility of research or external validation. Restrictions may be applied to sensitive data for privacy preservation.
