# Supplementary material for: Laboratory prognostic factors for the long-term survival of multiple system atrophy
Source: NPJ Parkinsons Dis. 2022 Oct 27;8:141. doi: 10.1038/s41531-022-00413-9 (PMC9613998; doi:10.1038/s41531-022-00413-9)
Supplement: Supplementary file 1 — Supplementary materials [file 41531_2022_413_MOESM1_ESM.pdf]

**Supplementary Table 1: Collected blood biomarkers in MSA**

| Laboratory markers | Number of available cases (%) | Mean value (standard deviation | Time from the onset to laboratory test (years) | Time from first visit to lab test (years) |
|---|---|---|---|---|
| 'WBC (count/ul)' | 447 (69) | 8765.08 (1482.38) | 4.18 (2.78) | 1.63 (1.24) |
| 'ANC (count/ul)' | 433 (67) | 4350.19 (2749.90) | 4.20(2.85) | 1.63(1.29) |
| ‘Lymphocyte (count/ul)’ | 433 (67) | 2578.63 (968.71) | 4.20(2.85) | 1.63(1.29) |
| 'Hb (g/dL)' | 438 (67) | 13.07 (1.59) | 4.19(2.82) | 1.61(1.23) |
| 'RDW' | 465 (72) | 12.99(0.96) | 4.12(2.73) | 1.67(1.39) |
| 'PLT (count/ul)' | 438 (67) | 228.34(63.51) | 4.19(2.82) | 1.63(1.25) |
| 'Protein (g/dL)'' | 437 (67) | 7.03(0.56) | 4.16(2.81) | 1.59(1.20) |
| 'Albumin (g/dL)'' | 443 (68) | 4.08(0.43) | 4.18(2.82) | 1.60(1.20) |
| 'Cholesterol (g/dL)'' | 450 (69) | 175.54 (38.95) | 4.14(2.80) | 1.57(1.17) |
| 'LDL (mg/dL)' | 245 (38) | 108.25 (35.23) | 3.93(2.64) | 1.68(2.31) |
| 'HDL(mg/dL)' | 232 (36) | 48.22 (14.41) | 3.91(2.66) | 1.54(1.16) |
| 'hs-CRP' | 341 (52) | 1.75 (4.92) | 4.40(2.91) | 1.72(1.47) |
| 'ESR (mm/hr)' | 290 (45) | 20.56 (19.71) | 3.99(2.63) | 1.60(1.28) |
| 'Uric Acid (mg/dL)' | 435 (67) | 4.82 (1.48) | 4.27(2.78) | 1.60(1.20) |
| 'Sodium (mEq/L )' | 414 (64) | 140.36 (3.25) | 4.27(2.79) | 1.63(1.25) |
| 'Potassium (mEq/L )' | 414 (64) | 4.22 (0.44) | 4.29(2.81) | 1.64(1.26) |
| 'Chloride (mEq/L)' | 414 (64) | 103.55 (3.57) | 4.20(2.82) | 1.64(1.25) |
| 'BUN (mg/dL)' | 414 (64) | 16.66 (6.54) | 4.20(2.82) | 1.62(1.22) |
| 'Creatinine (mg/dL)' | 414 (64) | 0.87 (0.31) | 4.46(2.82) | 1.62(1.22) |
| 'eGFR (mL/min/1.73 $m^2$)' | 403 (62) | 87.52(26.55) | 4.19(2.87) | 1.74(1.33) |
| 'Calcium (mg/dL)' | 437 (67) | 9.09(0.48) | 4.19(2.82) | 1.59(1.20) |
| 'Phosphorus (mg/dL)' | 436 (67) | 3.48(0.59) | 4.18(2.82) | 1.60(1.21) |
| 'GOT (Unit/L)' | 438 (67) | 22.35(12.53) | 4.18(2.82) | 1.58(1.18) |
| 'GPT' (Unit/L) | 438 (67) | 19.65(16.23) | 4.18(2.82) | 1.58(1.18) |
| VitB12 (pg/mL) | 345 (53) | 811.79(1091.83) | 3.42(2.82) | 1.30(0.99) |
| Orthostatic BP | 383 (59) | sBP drop 16.12 (20.11)<br>dBP drop 8.22 (12.03) | 3.99(2.51) | 1.84(0.88) |

All scores are shown as number or the mean (standard deviation).

Abbreviation, WBC: white blood cell count, ANC: absolute neutrophil count, Hb: hemoglobin, RDW: red cell

distribution width, PLT: platelet count, LDL: low density lipoprotein, HDL: high density lipoprotein, serum uric acid, serum, BUN: Blood urea nitrogen, creatinine, eGFR: estimated Glomerular filtration rate, AST: aminotransferase, ALT: alanine aminotransferase, CRP: C-reactive protein, ESR: erythrocyte sedimental rate, ul: microliter, dL:deciliter, mEq: milliequivalent, pg: picogram

**Supplementary Table 2. Mean (standard deviation) value of baseline laboratory markers in deceased and alive MSA patients.**

| Laboratory markers | Deceased MSA patient | Alive MSA patients | p-value |
|---|---|---|---|
| 'WBC (count/ul)' | 9658.97 (1637.48) | 7487.39 (1220.16) | 0.13 |
| 'ANC (count/ul)' | 4658.08(3101.97) | 3891.89(2044.50) | 0.0044 |
| ‘Lymphocyte (count/ul)’ | 2775.02(1111.75) | 2282.16(768.95) | 0.10 |
| 'Hb (g/dL)' | 13.02 (1.72) | 13.14(1.39) | 0.43 |
| 'RDW' | 13.13(1.00) | 12.79(0.86) | 0.00017 |
| 'PLT (x1000 count/ul)' | 224.08(64.88) | 234.51(61.12) | 0.091 |
| 'Protein (g/dL)'' | 6.97(0.59) | 7.11(0.51) | 0.016 |
| 'Albumin (g/dL)'' | 4.02(0.47) | 4.18(0.35) | $6.33x10^{-5}$ |
| 'Cholesterol (g/dL)'' | 174.59(39.29) | 176.99(38.49) | 0.52 |
| 'LDL (mg/dL)' | 109.14(34.01) | 107.21(36.74) | 0.67 |
| 'HDL(mg/dL)' | 48.68(14.88) | 47.36(13.54) | 0.51 |
| 'hs-CRP' | 2.09(5.09) | 1.28(4.66) | 0.13 |
| 'ESR (mm/hr)' | 19.94(19.48) | 21.71(20.20) | 0.47 |
| 'Uric Acid (mg/dL)' | 4.80(1.48) | 4.84(1.49) | 0.76 |
| 'Sodium (mEq/L)' | 140.23(3.52) | 140.55(2.83) | 0.31 |
| 'Potassium (mEq/L)' | 4.21(0.45) | 4.23(0.43) | 0.73 |
| 'Chloride (mEq/L)' | 103.42(4.01) | 103.73(2.85) | 0.38 |
| 'BUN (mg/dL)' | 16.85(6.76) | 16.37(6.22) | 0.44 |
| 'Creatinine (mg/dL)' | 0.89(0.34) | 0.83(0.25) | 0.030 |
| 'eGFR (mL/min/1.73 $m^2$)' | 87.23(28.26) | 87.88(24.22) | 0.81 |
| 'Calcium (mg/dL)' | 9.03(0.51) | 9.17(0.41) | 0.0026 |
| 'Phosphorus (mg/dL)' | 3.48(0.61) | 3.48(0.56) | 0.93 |
| 'GOT (Unit/L)' | 22.24(12.87) | 22.51(12.06) | 0.83 |
| 'GPT' (Unit/L) | 19.37(16.79) | 20.06(15.41) | 0.66 |
| VitB12 (pg/mL) | 852.9(988.67) | 752.31(1227.10) | 0.40 |
| Systolic BP drop | 18.72(21.98) | 13.54(17.74) | 0.011 |
| Diastolic BP drop | 10.01(13.48) | 6.43(10.09) | 0.0035 |

**Supplementary Table 3. Baseline laboratory markers that were collected within a year from initial visit to the clinic.**

| Laboratory markers | Available cases (%) | Mean value (standard deviation | Time from the onset to laboratory test (years) |
|---|---|---|---|
| 'WBC (count/ul)' | 321 (49) | 8760.46 (1490.59) | 3.38 (2.17) |
| 'ANC (count/ul)' | 311 (48) | 4216.93 (2602.27) | 3.35(2.17) |
| ‘Lymphocyte (count/ul)’ | 311 (48) | 2682.36 (967.03) | 3.35(2.17) |
| 'Hb (g/dL)' | 315 (48) | 13.18(1.49) | 3.38(2.16) |
| 'RDW' | 340 (52) | 12.95(0.86) | 3.36(2.21) |
| 'PLT (count/ul)' | 314 (48) | 227.95(65.34) | 3.38(2.21) |
| 'Protein (g/dL)'' | 317 (49) | 7.08(0.53) | 3.33(2.17) |
| 'Albumin (g/dL)'' | 319 (49) | 4.15(0.39) | 3.39(2.23) |
| 'Cholesterol (g/dL)'' | 330 (51) | 179.29(39.39) | 3.35(2.19) |
| 'LDL (mg/dL)' | 245 (38) | 108.25(35.23) | 3.93(2.64) |
| 'HDL(mg/dL)' | 174 (27) | 48.69(14.86) | 3.24(2.28) |
| 'hs-CRP' | 238 (37) | 1.82(5.57) | 3.59(2.50) |
| 'ESR (mm/hr)' | 212 (33) | 19.41(18.51) | 3.26(2.14) |
| 'Uric Acid (mg/dL)' | 314 (48) | 4.94(1.45) | 3.42(2.23) |
| 'Sodium (mEq/L)' | 294 (45) | 140.58(3.10) | 3.53(2.33) |
| 'Potassium (mEq/L)' | 293 (45) | 4.23(0.44) | 3.50(2.32) |
| 'Chloride (mEq/L)' | 292 (45) | 103.90(3.36) | 3.48(2.30) |
| 'BUN (mg/dL)' | 315 (48) | 16.41(6.22) | 3.36(2.22) |
| 'Creatinine (mg/dL)' | 314 (48) | 0.88(0.28) | 3.37(2.22) |
| 'eGFR (mL/min/1.73 $m^2$)' | 268 (41) | 85.37(22.97) | 3.56(2.35) |
| 'Calcium (mg/dL)' | 318 (49) | 9.14(0.46) | 3.43(2.26) |
| 'Phosphorus (mg/dL)' | 315 (48) | 3.52(0.58) | 3.39(2.21) |
| 'GOT (Unit/L)' | 319 (49) | 22.75(12.82) | 3.39(2.23) |
| 'GPT' (Unit/L) | 318 (49) | 20.58(16.48) | 3.38(2.21 |
| VitB12 (pg/mL) | 296 (46) | 747.41(774.82) | 3.03(2.02) |
| Orthostatic BP | 179 (28) | sBP drop 16.37 (19.88)<br>dBP drop 8.68 (12.50) | 3.06(1.64) |

**Supplementary Figure 1. Hazard ratio for each laboratory biomarkers in prediction of mortality in MSA-P and MSA-C.**

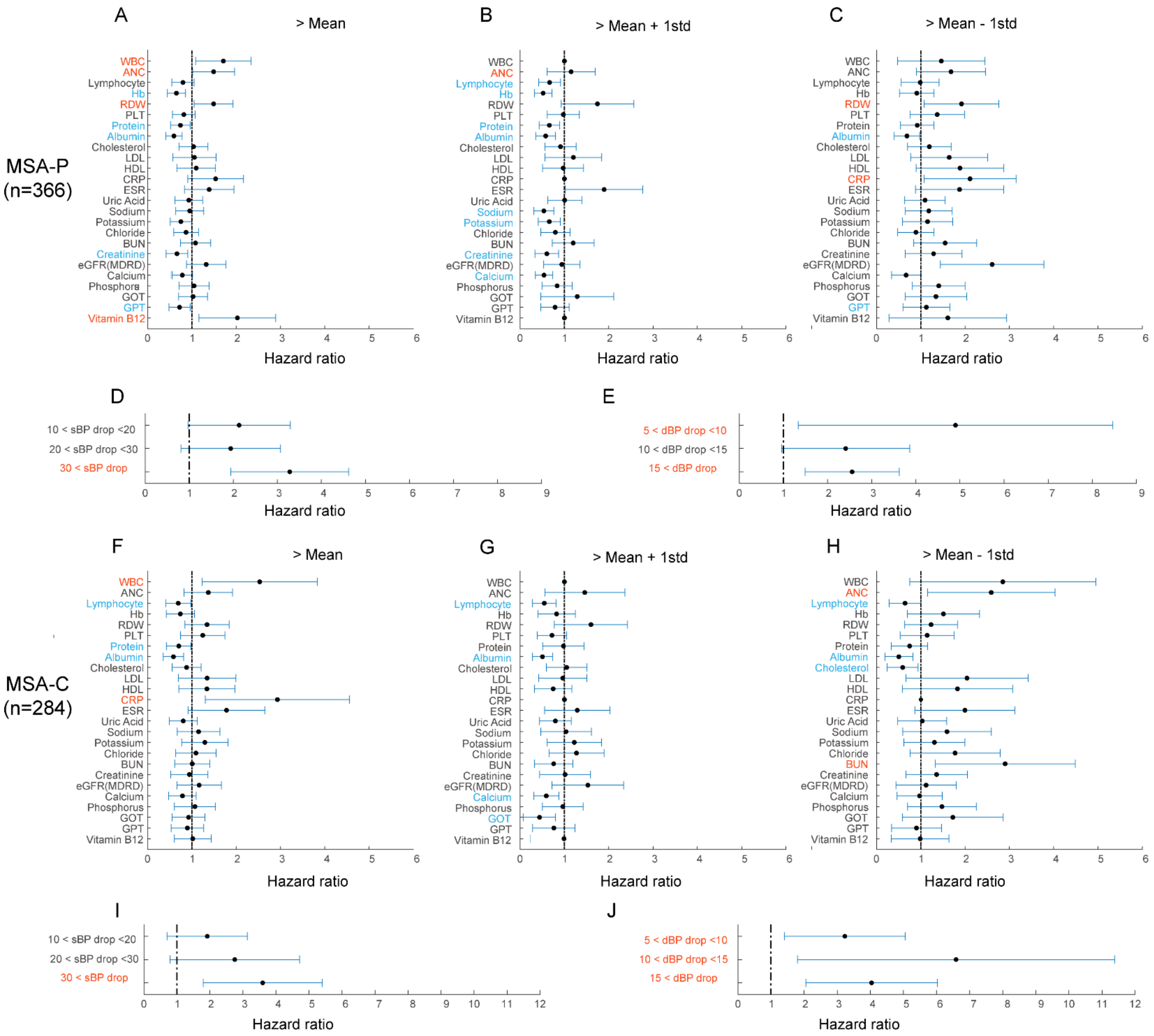

**A-C.** Bar graph of hazard ratio±95% confidence interval from each blood laboratory marker with a cut-offs(> mean, > mean + 1std, >Mean – 1std) predicting mortality from longitudinal follow-up of MSA-P patients. The hazard ratios with statistical significance ($p<0.05$) were marked as red (positive) or light blue (negative). A vertical dotted line denotes a hazard ratio of 1. **D-E.** Bar graph of hazard ratio ± 95% confidence interval of systolic blood pressure (sBP) drop (D) and diastolic blood pressure (dBP) drop in orthostatic blood pressure test. Hazard ratio for sBP was calculated comparing MSA groups of [10 < sBP drop <20 versus sBP < 10] and [20 < sBP drop <30 versus sBP <20], [30 < sBP versus sBP <30]. Hazard ratio for dBP was calculated comparing MSA groups of [5 < dBP drop <10 versus dBP < 5] and [10 < dBP drop <15 versus dBP <10], [15 < dBP versus dBP <15]. **F-H.** Bar graph of hazard ratio±95% confidence interval from each blood laboratory marker with a cut-offs(> mean, > mean + 1std, >Mean – 1std) predicting mortality from longitudinal follow-up of MSA-C patients. The hazard ratios with statistical significance ($p<0.05$) were marked as red (positive) or light blue (negative). A vertical dotted line denotes a hazard ratio of 1. **I-J.** Bar graph of hazard ratio ± 95% confidence interval of systolic blood pressure (sBP) drop (D) and diastolic blood pressure (dBP) drop in orthostatic blood pressure test. Hazard ratio for sBP was calculated comparing MSA groups of [10 < sBP drop <20 versus sBP < 10] and [20 < sBP drop <30 versus sBP <20], [30 < sBP versus sBP <30]. Hazard ratio for dBP was calculated comparing MSA groups of [5 < dBP drop <10 versus dBP < 5] and [10 < dBP drop <15 versus dBP <10], [15 < dBP versus dBP <15].

Abbreviations, std: Standard deviation WBC: white blood cell count, ANC: absolute neutrophil count, Hb: hemoglobin, RDW: red cell distribution width, PLT: platelet count, LDL: low-density lipoprotein, HDL: high-density lipoprotein, CRP: C-reactive protein, ESR: erythrocyte sedimentation rate, BUN: Blood urea nitrogen, eGFR: estimated Glomerular filtration rate, GOT: glutamic oxaloacetic transaminase, GPT: glutamic pyruvic transaminase.

**Supplementary Figure 2. Hazard ratio for each laboratory biomarkers collected within a year from first visit in prediction of mortality in MSA.**

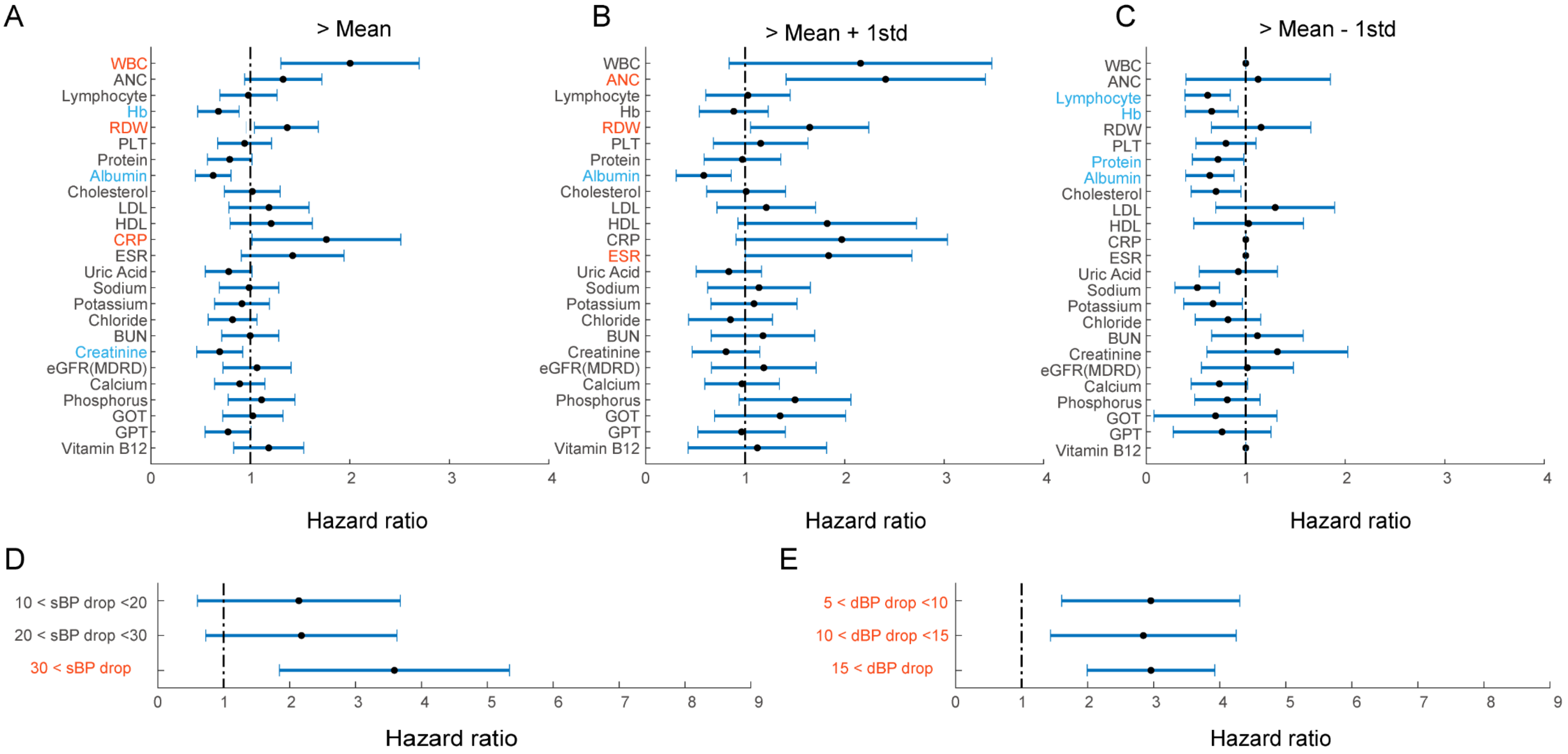


**A-C.** Bar graph of hazard ratio±95% confidence interval from each blood laboratory marker that was collected within a year from first visit with a cut-offs(> mean, > mean + 1std, >Mean – 1std) predicting mortality from longitudinal follow-up of MSA patients. The hazard ratios with statistical significance (p<0.05) were marked as red (positive) or light blue (negative). A vertical dotted line denotes a hazard ratio of 1. **D-E.** Bar graph of hazard ratio ± 95% confidence interval of systolic blood pressure (sBP) drop (D) and diastolic blood pressure (dBP) drop in orthostatic blood pressure test. Hazard ratio for sBP was calculated comparing MSA groups of [10 < sBP drop <20 versus sBP < 10] and [20 < sBP drop <30 versus sBP <20], [30 < sBP versus sBP <30]. Hazard ratio for dBP was calculated comparing MSA groups of [5 < dBP drop <10 versus dBP < 5] and [10 < dBP drop <15 versus dBP <10], [15 < dBP versus dBP <15].
